# Contribution of the Elastic Component and Venous Wall Arterialization in Patients with Venous Reflux

**DOI:** 10.3390/jpm12020260

**Published:** 2022-02-10

**Authors:** Miguel A. Ortega, Oscar Fraile-Martínez, Cielo García-Montero, Fernando Ruiz-Grande, Miguel Angel Álvarez-Mon, Jorge Monserrat, Luis G. Guijarro, Santiago Coca, Melchor Álvarez-Mon, Julia Bujan, Natalio García-Honduvilla, Miguel A. Sáez

**Affiliations:** 1 Department of Medicine and Medical Specialties, Faculty of Medicine and Health Sciences, University of Alcalá, 28801 Alcalá de Henares, Spain; oscarfra.7@hotmail.com (O.F.-M.); cielo.gmontero@gmail.com (C.G.-M.); maalvarezdemon@icloud.com (M.A.Á.-M.); jorge.monserrat@uah.es (J.M.); s.coca@uah.es (S.C.); mademons@gmail.com (M.Á.-M.); mjulia.bujan@uah.es (J.B.); natalio.garcia@uah.es (N.G.-H.); msaega1@oc.mde.es (M.A.S.); 2 Ramón y Cajal Institute of Healthcare Research (IRYCIS), 28034 Madrid, Spain; luis.gonzalez@uah.es; 3 Cancer Registry and Pathology Department, Hospital Universitario Principe de Asturias, 28801 Alcalá de Henares, Spain; 4 Service of Angiology and Vascular Surgery, Hospital Universitario de la Princesa, 28006 Madrid, Spain; fruizgrande@hotmail.com; 5 Unit of Biochemistry and Molecular Biology (CIBEREHD), Department of System Biology, University of Alcalá, 28801 Alcalá de Henares, Spain; 6 Immune System Diseases-Rheumatology and Oncology Service, University Hospital Príncipe de Asturias, CIBEREHD, 28801 Alcalá de Henares, Spain; 7 Pathological Anatomy Service, Central University Hospital of Defence-UAH, 28047 Madrid, Spain

**Keywords:** chronic venous disease (CVeD), venous reflux, elastic fibers, tropoelastin, venous arterialization

## Abstract

Chronic venous disease (CVeD) is defined as a set of disorders affecting the venous system mainly manifested in the form of varicose veins. CVeD is characterized by a sustained venous hypertension, leading to a plethora of functional and structural changes in the vein that may cause valve incompetence and pathologic reflux. In turn, venous reflux aggravates the venous hypertension and enhances the progression of CVeD into the most advanced stages. Previous studies have proposed that there are several alterations in the venous wall preceding the valve dysfunction and venous reflux. Besides, it has also been identified that young patients with CVeD present premature aging and changes in the venous wall composition that may be related to the presence of venous reflux. In this context, the aim of the present study is to examine the possible pathophysiological role of elastic fibers and their precursors in the venous wall of patients with reflux in comparison to those without reflux, considering the variable age in both groups (<50 years and ≥50 years). We performed immunohistochemical and quantitative polymerase chain reaction (PCR) in order to assess the protein and gene expression of tropoelastin, fibrillin-1, fibulins 4 and 5, lysyl oxidase and lysyl oxidase like 1, respectively. In parallel, we assessed the elastin content through histological techniques (orcein stain) in this group of patients. Our results show significant changes in elastic fibers and their precursors in young patients with pathologic reflux when compared with elder patients with reflux and young patients without reflux. These variations suggest that the venous system of young patients with venous reflux appears to present an enhanced dynamism and arterialization of the venous wall, which may be associated with a premature aging and pathological environment of the tissue.

## 1. Introduction

Chronic venous disease (CVeD) comprises a set of functional and structural disorders affecting the venous system, which is a highly prevalent but underestimated global concern [1]. The classification of CVeD is performed following the Clinical-Etiology-Anatomy-Pathophysiology (CEAP) criteria, considering the main clinical manifestations, causes, veins affected and pathophysiological mechanisms involved [2]. In this sense, varicose veins (VVs) are the most common clinical sign of CVeD, corresponding to the C2 classification. Because of the gravitational force and the differential blood pressure to overcome, the venous system located in the lower limbs is more prone to result in VVs. Approximately 25% of the population may suffer from VVs in the lower limbs, with an estimated incidence around 2% per year [3]. According to the Framingham study, the incidence of VVs is slightly superior in women than in men and it is frequently associated with the exposure to some risk factors like lower levels of physical activity, higher body mass index and systolic blood pressure or menopause [4]. If untreated, VVs may progress to more severe signs and symptoms encompassed in the term chronic venous insufficiency (CVI), ranging from edema (C3) and cutaneous manifestations (C4) to healed and active ulcerations (C5/C6) [5]. Besides, VVs and CVI, rather than being an esthetic or local concern, may also cause pain, heaviness and discomfort, and they may even be related with more serious consequences like deep venous thrombosis, some types of neoplasia or depression [6].

Regarding its pathophysiology, CVeD is classified by the presence of reflux, obstruction, a combination of both or due to unidentified mechanisms [2]. Some epidemiological data reflect that venous reflux may occur in 35% of the general population, affecting either superficial or deep veins [7]. The estimated annual incidence is of approximately 1% [8], and it seems that there is a positive association between age and venous reflux in the superficial veins [9]. Despite the fact that venous reflux is based on different mechanisms, it is recognized to play a central role in venous valve dysfunction, vascular inflammation and hemodynamic alterations, prominently with venous hypertension, which initiates and triggers all these pathophysiological mechanisms [5]. Accompanying these changes, there is a profound remodeling of the venous wall preceding the venous reflux, aggravating the venous hypertension, venous dilation and participating in the progression of VVs to CVI [10].

The changes of the venous wall structure in patients with CVeD have led to some authors to hypothesize that the veins may suffer a process of “arterialization”, characterized by an altered behavior and composition of the cellular and extracellular components in the venous wall [11]. In this sense, previous studies have found that there is an important remodeling of collagen and elastic fibers in VVs probably involved in the process of arterialization, dilation and distensibility of the venous wall [12]. The role of the collagen components in venous reflux and VV pathogenesis has received greater attention during the recent years; however, to our knowledge, the elastic component and the molecular basis of its alterations have been barely studied. Thus, the aim of this work is the analysis and implication of the elastic extracellular matrix in patients with VVs, and to consider diagnosed reflux in comparison to VVs with no diagnosis of reflux. Furthermore, we intend to evaluate possible differences in young (<50 years) versus elder (<50) individuals, in order to identify potential pathophysiological mechanisms implicated in the pathogenesis of CVeD process in younger patients. Herein, we will determine the genic and protein expression by real-time PCR (RT-qPCR) and immunohistochemistry, respectively, of different components of the elastic matrix including elastin, tropoelastin (TE), fibrilin-1 (FBN1), fibulin 4 (FBLN4), FBLN5 and the enzymes lysyl oxidase (LOX) and lysyl oxidase like 1 (LOXL1).

## 2. Patients and Methods

### 2.1. Study Population

In this study, 110 patients were divided according to their age (cut-off at 50 years) and the presence of pathologic reflux (R) or absence (NR) of a clinical diagnosis of venous reflux (an indicator of venous system valve incompetence). The following study groups were established: NR, *n* = 29, 50.0 (31.0–79.0) years; NR < 50, *n* = 13, 38.0 (31.0–48.0) years; NR ≥ 50, *n* = 16, 62.5 (50.0–79.0) years; R, *n* = 81, 51.0 (22.0–79.0) years; R < 50, *n* = 32, 35.0 (22.0–48.0) years; and R ≥ 50, *n* = 49, 62.0 (50.0–79.0) years. The study cohort was selected according to the following criteria:

Inclusion criteria: men and women men diagnosed with CVeD with or without venous reflux in the great saphenous vein, Body mass index (BMI) ≤ 25, commitment to follow-up during pre- and postoperative periods, signed informed consent and permission for tissue collection to develop gene and histopathological studies.

Exclusion criteria: patients with venous malformations or arterial insufficiency, patients who did not provide their medical history, patients with cardiovascular alterations (e.g., infectious diseases, diabetes, dyslipidemia, hypertension), toxic habits (tobacco (≥1 cigarette a day), alcohol (≥1 unit a day) or drugs (cannabis, heroin, cocaine, amphetamines)) and patients who rejected to commit to follow-up.

The study was approved by the Ethics Committee of Clinical Investigations of the Central University Hospital of Defense Gómez-Ulla-UAH (03-37/17, date of approval: 03 March 2017). This study was conducted in agreement with the basic ethical principles of autonomy, beneficence, nonmaleficence and distributive justice. The study protocol was in line with the standards of Good Clinical Practice and the principles set out in the most recent Declaration of Helsinki (2013) and Oviedo Convention (1997). The patients were duly informed, and each was asked to provide written informed consent.

### 2.2. Diagnosis

The patients were evaluated using a 7.5 Mz M Turbo Transducer Echo-Doppler (SonoSite, Bothell, WA, USA). The lower limb examination was performed in the standing position, with one leg in external rotation and the contralateral leg supported. The exam included the great saphenous axis from the inguinal region to the femoral ankle and vein. The saphenous vein and popliteal vein were also evaluated in the standing position, with the patient facing away from the examiner and with the leg unloaded, as described by Ortega et al. [13]. All patients were classified as CEAP C1 or higher (Table 1). Saphenectomy was indicated in all patients with R, defined as a reflux time longer than 0.5 s. In the patients with NR, vascular compressive syndrome was the surgical indication.

### 2.3. Collection of Vein Tissue Samples

The total saphenous arch was removed after saphenectomy for further analysis and placed into two sterile tubes. The first tube contained Minimum Essential Medium (MEM) with 1% antibiotic/antimycotic (both from Thermo Fisher Scientific, Waltham, MA, USA) and the second had RNAlater^®^ Solution (Ambion, Austin, TX, USA). The samples were transported under refrigeration within four hours of collection and then in the laboratory, and they were processed under sterile conditions under a Telstar AV 30/70 Müller 220 Hz class II laminar flow hood (Group Telstar SA, Terrassa, Spain). The samples preserved in MEM were destined for histological studies; they were washed/hydrated several times with MEM without antibiotics to remove blood cells and cut into fragments that were placed in F13 fixative (60% ethanol, 20% methanol, 7% polyethylene glycol and 13% distilled H_2_O) for future studies. The samples placed in RNAlater^®^ were stored in 1 mL of this solution at −80 °C until further processing for gene expression analysis.

### 2.4. Immunohistological Studies

After fixation (10 days), the samples were dehydrated and processed following standardized protocols [14]. Then, paraffin blocks were made thanks to the use of molds. Once the paraffin solidified, a rotation microtome HM 350 S (Thermo Fisher Scientific, Waltham, MA, USA) was used to cut 5 μm thick sections, which were stretched in a hot water bath and collected on glass slides coated with 10% poly-L-lysine to facilitate tissue bonding. The avidin-biotin complex (ABC) method was employed to detect antigen-antibody reactions using specific primary (Table 2), secondary antibodies (Table 2) and chromogen peroxidase or alkaline phosphatase according to the protocol published by Ortega et al. [15]^.^ The slides were then incubated with the avidin-peroxidase conjugate ExtrAvidin^®^-Peroxidase (1/200; Sigma-Aldrich, St. Louis, MO, USA) for 1 h at room temperature. Incubation with the chromogenic substrate diaminobenzidine (Vector^®^ DAB Kit, SK-4100; Vector, Burlingame, CA, USA) for 15 min permitted to see avidin-peroxidase conjugate signals (the signal was monitored under the microscope). The chromogenic substrate was prepared just before development—5 mL of distilled water, two drops of buffer, four drops of DAB and two drops of hydrogen peroxide, according to Vector^®^ DAB Kit, allowing for a brown staining. For all immunohistochemical studies, sections of the same tissue incubated in blocking solution without the primary antibody were used as negative controls.

### 2.5. Orcein Methods

For orcein staining, the samples collected in the glass slide were deparaffinized for 30 min in xylol (PanReac AppliChem, Barcelona, Spain) and subsequently rehydrated by passing them in alcohols of decreasing concentrations (100%, 96% and 70%), until they were completely hydrated in distilled water. After rehydration, the sections of the samples were stained with alcoholic orcein for 30 min and then washed with distilled water for 30 min. Afterwards, the samples were immersed in 96% alcohol for 5 min, 100% alcohol for 15 min and acid alcohol for 2–10 min to achieve discoloration of the bottom. Subsequently, they were washed with water for 10 min, contrasted with Carazzi hematoxylin for 20 min and washed again in running water for 10 min. The last steps were dehydration in 96% alcohol for 5 min, dehydration in 100% alcohol for 5 min, xylol clearance for 10 min and Cytoseal™ mounting (Epredia™ 8310-4, Thermo Fisher Scientific A., Waltham, MA, USA). The use of this technique evidences the presence of the elastic fibers in a brown color [16].

### 2.6. Analysis of Gene Expression Using qRT-PCR

RNA was extracted from the samples collected in RNAlater^®^ using the guanidine-phenol-chloroform isothiocyanate method [17], and RNA samples were always kept on ice. (1) To synthesize complementary DNA (cDNA) via reverse transcription, RNA samples were diluted to 50 ng/μL; then, 4 μL of diluted RNA was mixed with 4 μL of oligo-dT solution at 0.25 µg/µL (Thermo Fisher Scientific A., Waltham, MA, USA) and incubated at 65 °C for 10 min in a dry bath (AccuBlock™, Labnet International Inc., Edison, NJ, USA) to denature the RNA. (2) Next, the samples were put on ice, and 10 μL of a reverse transcription mixture was added to each sample in agreement with established protocols [18]. (3) Reverse transcription was performed to synthesize cDNA using a G-Storm GS1 thermocycler (G-Storm, Somerset, UK) at 37 °C for 1 h and 15 min, 70 °C for 15 min (leading to denaturation of the reverse transcriptase enzyme) and then a gradual decrease to 4 °C. (4) To verify the absence of genomic DNA contamination in the total RNA samples, negative control samples were run in parallel; in these samples, M-MLV RT was replaced with DNase- and RNase-free water. (5) The generated cDNA was diluted to 1:20 using DNase- and RNase-free water and stored at −20 °C until use. (6) qPCR was performed to quantify the levels of cDNA of our genes of interest. (7) Specific primers were designed de novo for the studied genes (Table 3) using Primer-BLAST (National Center for Biotechnology Information, Bethesda, MD, USA) and AutoDimer [18]. Glyceraldehyde 3-phosphate dehydrogenase (*GAPDH*) gene was used to normalize the results. (8) qPCR was performed in a StepOnePlus™ System (Thermo Fisher Scientific, Waltham, MA, USA), and the relative standard curve method was used. For each sample, 5 μL of the sample diluted to 1:20 was mixed with 10 μL of iQ™ SYBR^®^ Green Supermix (Bio-Rad Laboratories, Hercules, CA, USA), 1 μL of forward primer, 1 μL of reverse primer and 3 μL of DNase- and RNase-free water, for a total reaction volume of 20 μL in a 96-well MicroAmp^®^ plate (Thermo Fisher Scientific, Waltham, MA, USA). (9) Fluorescence was detected at the end of each amplification cycle and at each step of the dissociation curve. (10) Finally, the data obtained for each gene were compared to a standard curve based on serial dilutions of a mixture of the study samples included in each plate according to expression of *GADPH*. Gene expression units are expressed as the relative quantity mRNA (RQ). All the assays were carried out in duplicate.

### 2.7. Statistical Analysis and Interpretation of the Results

GraphPad Prism^®^ 5.1 software (GraphPad Software, San Diego, CA, USA) was used with Mann–Whitney U test were used for our statistical analysis. The data are presented as the median with interquarile range (IQR), and significance was established at *p* < 0.05 (*), *p* < 0.005 (**) and *p* < 0.0001 (***). In total, 5 sections and 10 fields per section were randomly selected and examined for each patient. Patients were characterized as positive when the average marked area in the sample analyzed was greater or equal to 5% of the total area, following the anatomical-pathological protocol of Cristóbal et al. [19]. This procedure is a minimal modification of the immunoreactive score (ISR score). The preparations were studied under a Zeiss Axiophot light microscope (Carl Zeiss, Oberkochen, Germany) equipped with an AxioCam HRc digital camera (Carl Zeiss, Oberkochen, Germany).

## 3. Results

### 3.1. Young Patients with Venous Reflux Show an Increase in Elastic Fibers

**Figure 1:**
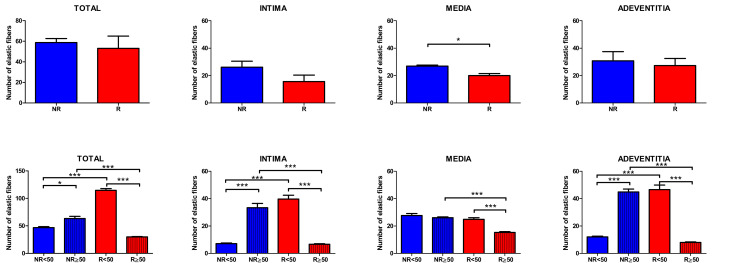
Quantification of the number of elastic fibers in the venous wall and in its three tunics of the different study patients. * *p* < 0.05, *** *p* < 0.0001.

**Figure 2:**
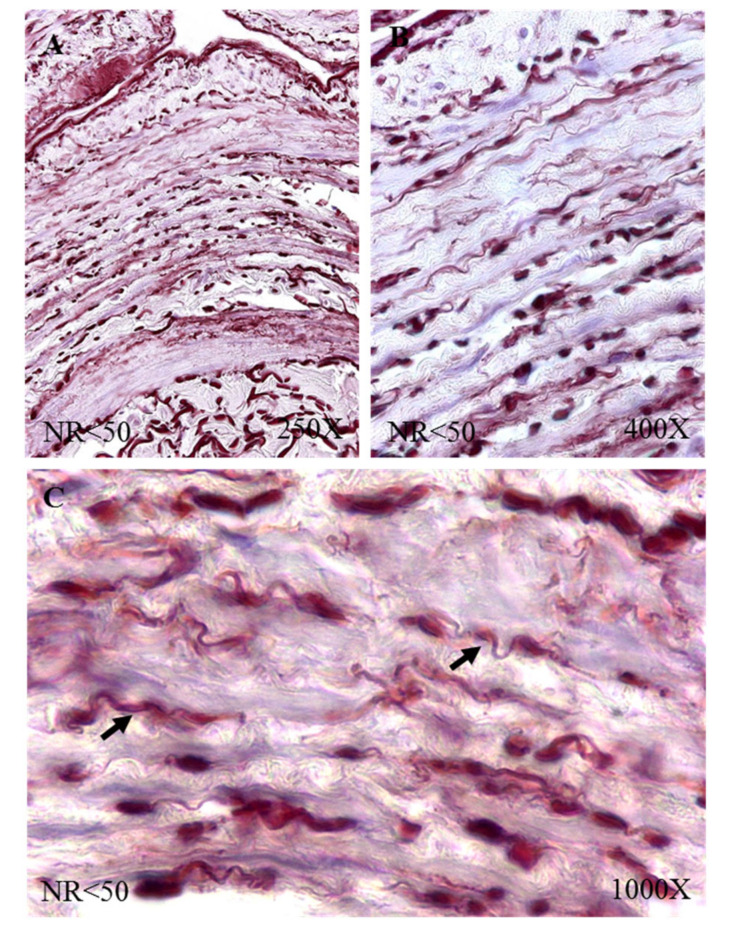
Images showing the elastic fibers in patients with NR < 50 (**A**–**C**).

**Figure 3:**
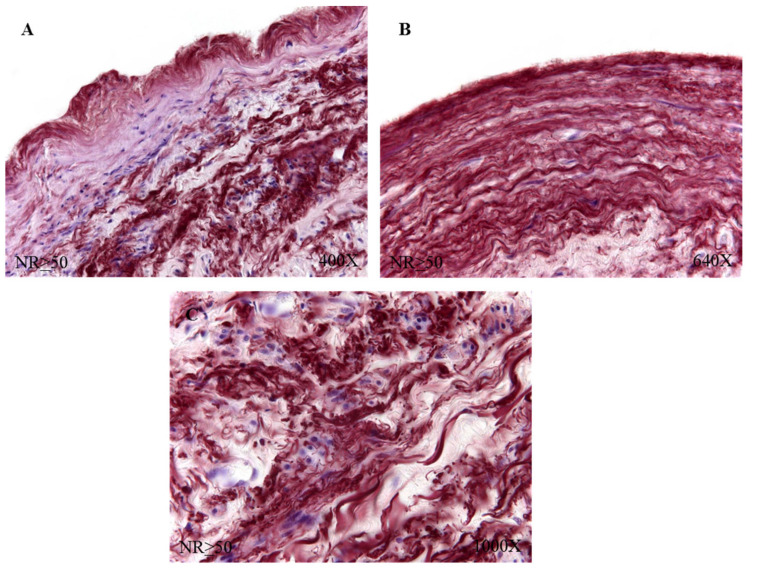
Images showing the elastic fibers in patients with NR ≥ 50 (**A**–**C**).

**Figure 4:**
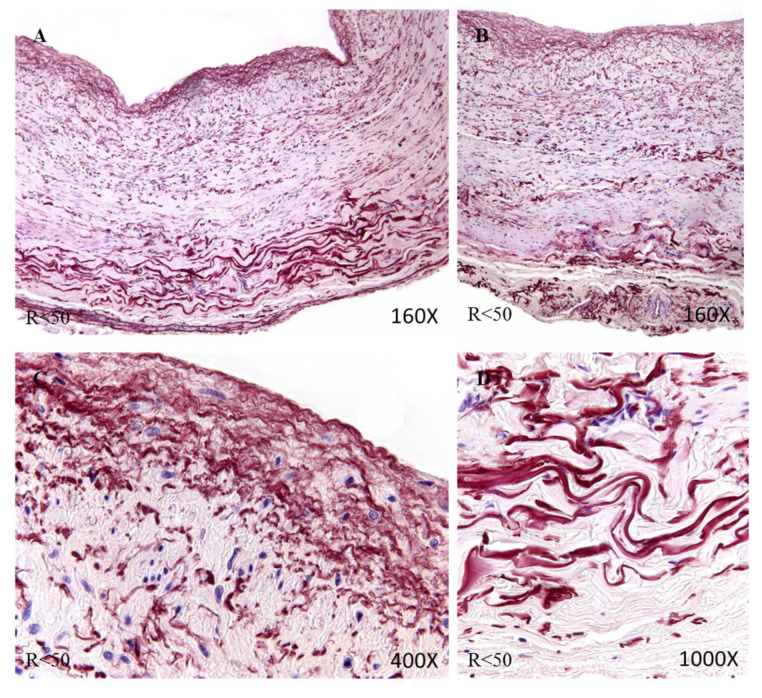
Images showing the elastic fibers in patients with R < 50 (**A**–**D**).

**Figure 5:**
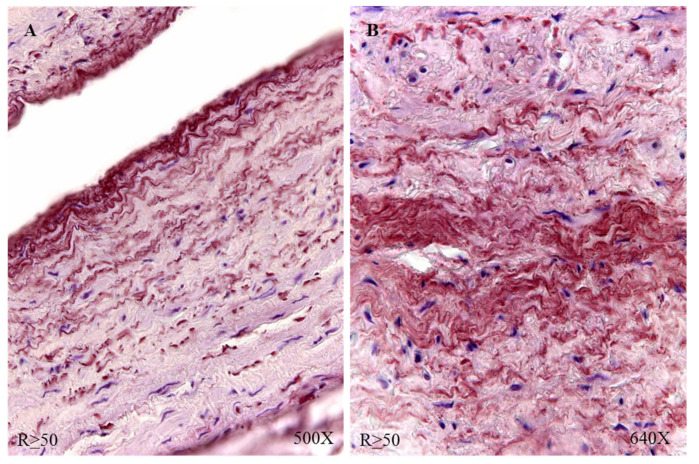
Images showing the elastic fibers in patients with R ≥ 50 (**A**,**B**).

The study of the elastic component was carried out by visualizing the elastic fibers in tissue samples. This study revealed differences in the number of elastic fibers depending on the presence or absence of venous reflux and the age of the patients. In global terms, we observed how the R patients had a statistically significant decrease in the number of elastic fibers in the tunica media of the vein wall (Figure 1 and Table 4). Considering the age factor, it was possible to describe how the NR ≥ 50 and R < 50 have the highest number of elastic fibers that were statistically significant (Figure 1 and Table 4). This fact was kept in the intimal and adventitial tunics (Figure 1, Figure 2, Figure 3, Figure 4 and Figure 5 and Table 4).

### 3.2. Patients with Venous Reflux Show Increased Expression of Tropoelastin

**Figure 6:**
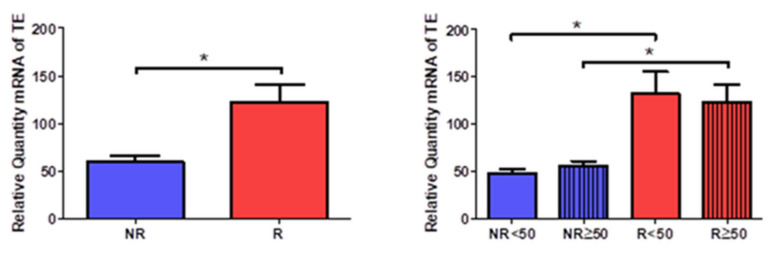
Levels of mRNA of the gene for tropoelastin quantified by RT-qPCR of patients without reflux (NR) and with reflux (R), as well as by their ages. * *p* < 0.05.

Tropoelastin gene expression analysis showed a statistically significant increase in R patients (NR = 59.129 ± 14.655, R = 122.576 ± 54.530, * *p* < 0.05, Figure 6). A significant increase was observed in patients R < 50 compared to patients NR < 50; this relationship of significance was maintained when comparing patients R > 50 and NR > 50 (NR < 50 = 47.599 ± 6.596, NR > 50 = 55.950 ± 7.906, R < 50 = 131.753 ± 53.041, R > 50 = 122.623 ± 40.703, * *p* < 0.05, Figure 6).

Protein expression by immunohistochemical techniques of tropoelastin revealed that the largest number of patients with positive immunodetection were in the venous reflux group: 91.34% compared to 65.52% of the NR patients. Taking into account the age factor, we observed how patients R < 50 had the highest percentage of expression (NR < 50 = 61.54%, NR > 50 = 68.75%, R < 50 = 93.75%, R > 50 = 89.80%).

**Figure 7:**
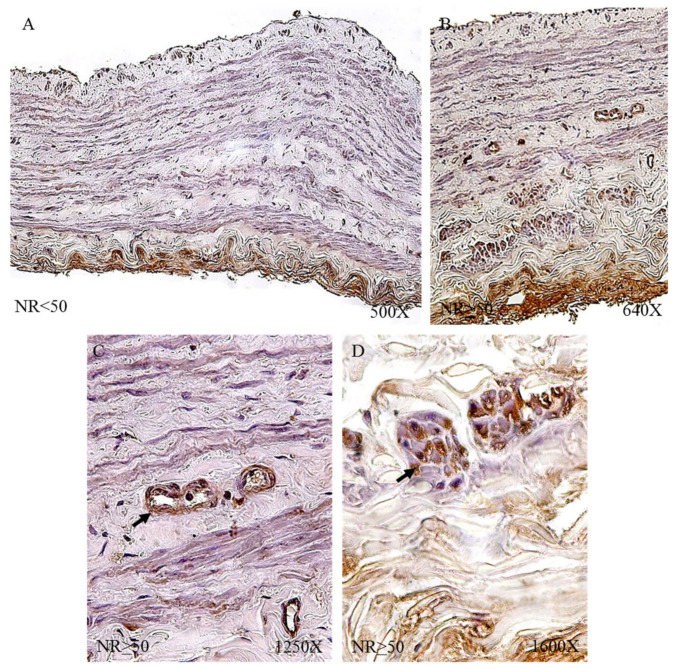
Images showing tropoelastin protein expression in patients with NR < 50 (**A**) and NR > 50 (**B**–**D**).

**Figure 8:**
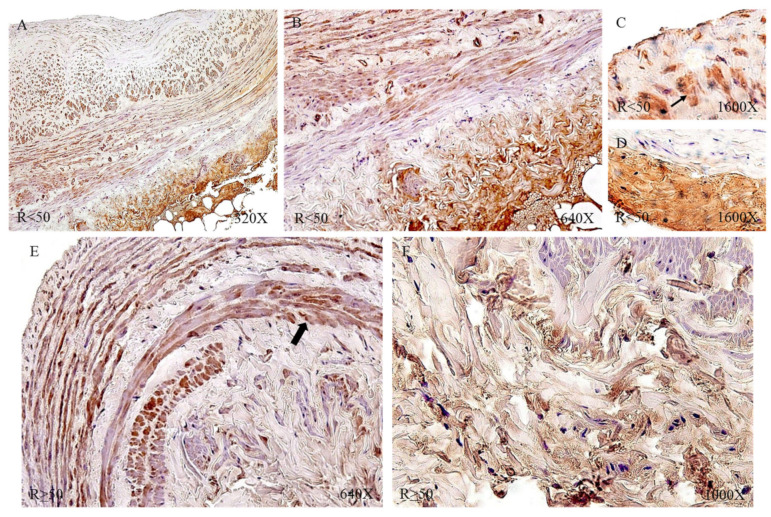
Images showing tropoelastin protein expression in patients with R < 50 (**A**–**D**) and R >50 (**E**,**F**).

The immunohistochemical studies of protein expression showed that, in the case of patients with NR < 50, the tropoelastin protein was located mainly in the adventitial layer, presenting a low-to-moderate intensity close to a mean point (Figure 7A). NR > 50 showed an expression located in the tunica media and adventitia of the vein wall (Figure 7B) and could be detected in blood capillaries (Figure 7C) and smooth muscle bundles (Figure 7D). The R < 50 patients presented a high intensity in the three tunics of the vein (Figure 8A,B). Tropoelastin was located with great intensity in smooth muscle fibers and blood capillaries, as well as in endothelial cells (Figure 8C). In the case of the adventitial tunic, a high-intensity expression was observed that occupied the entire vein tunic (Figure 8D). In the case of patients R > 50, a similar protein expression was observed (Figure 8E,F).

### 3.3. Young Patients with Venous Reflux Show Increased Levels of Fibulin 5 Expression

**Figure 9:**
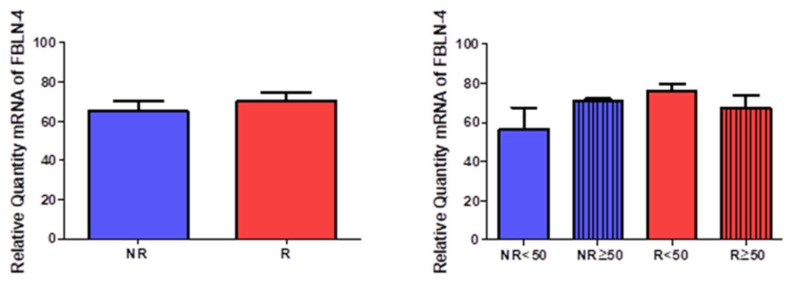
Expression levels of the gene for fibulin 4 quantified by RT-qPCR of patients without reflux (NR) and with reflux (R), as well as by their ages.

The quantification of the gene expression for fibulin 4 (FBLN-4) did not show data statistically between the study groups (NR = 65.348 ± 11.364, R = 70.181 ± 10.058, Figure 9). A slight trend towards increased expression can be observed in patients with venous reflux. The distribution of individuals according to age allows us to observe how the greatest expression occurs in patients R < 50 (NR < 50 = 56.528 ± 15.820, NR > 50 = 71.228 ± 1.871, R < 50 = 76.110 ± 5.054, R > 50 = 67.217 ± 12.654, Figure 9).

**Figure 10:**
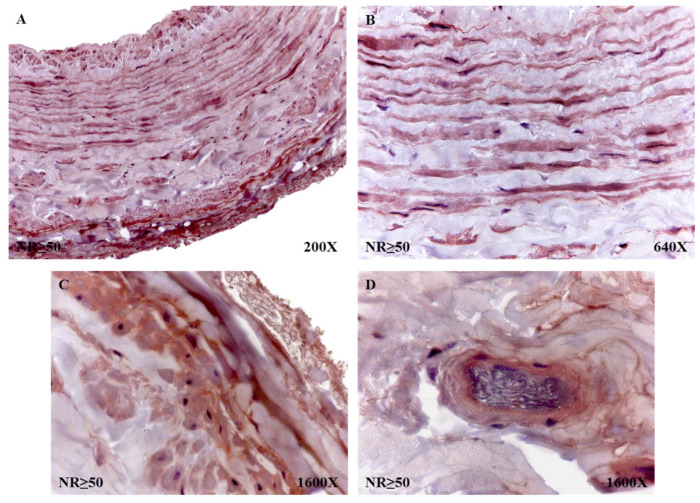
Fibulin 4 protein expression in patients without reflux older than or equal to 50 years (NR ≥ 50). Fibulin 4 expression is shown in all three tunics (**A**), smooth muscle fibers (**B**), detail of elastic fibers (**C**) and blood capillary (**D**).

The percentages of protein expression corroborated the trends observed in the molecular studies, with 58.63% for NR patients and 75.31% for patients with R. The percentages of said expression, taking into account that the age of the individuals was 53 years old, was 85% in NR < 50 patients and 62.50% in NR > 50 patients. In the case of the R patients, these percentages of protein expression rose to 90.63% in R < 50 and 65.31% in R > 50. The histopathological study of the immunohistochemical expression of fibulin 4 revealed how said expression remained similar in all study groups. However, it is noteworthy how the patients with NR > 50 had a high expression in the entire wall of the vein (Figure 10A). In the smooth muscle fibers, it was visualized how they were marked for said protein (Figure 10B). When studying the adventitial tunic, it was observed how the elastic fibers had reactivity for the reported protein (Figure 10C). In the entire vein wall, blood capillaries with positive expression for fibulin 4 were arranged (Figure 10D).

**Figure 11:**
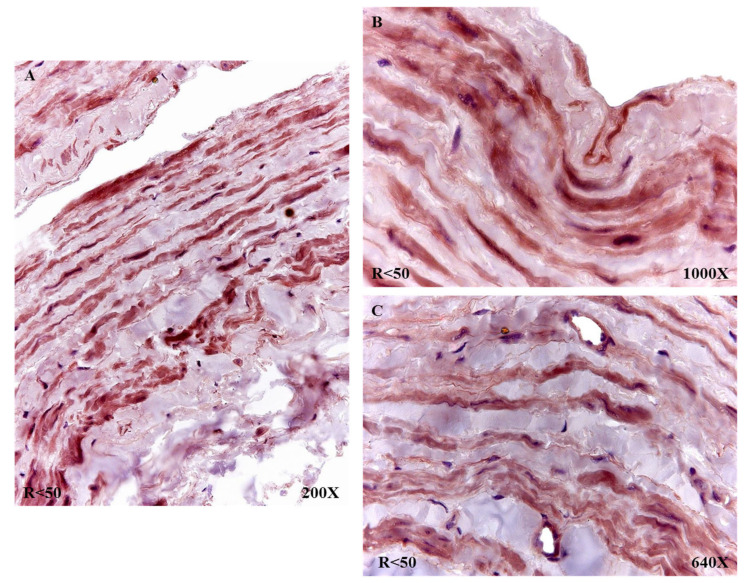
Fibulin 4 protein expression in patients with reflux younger than 50 years (R < 50). Fibulin 4 expression is shown in all three tunics (**A**), smooth muscle fibers (**B**), detail of elastic fibers and blood capillary (**C**).

In the case of patients R < 50, a similar trend was observed in terms of the expression of fibulin 4, with the entire venous wall reactive (Figure 11A). It stands out how these patients presented an intense intensity of expression in the vicinity of the insertion of what were venous valves (Figure 11B). All the smooth muscle fibers and blood capillaries were equally reactive as in the rest of the study patients (Figure 11C).

**Figure 12:**
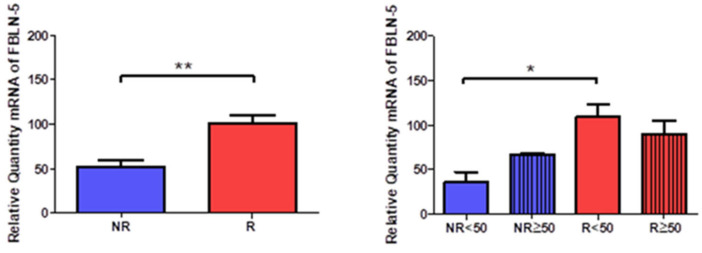
Expression levels of the gene for fibulin 5 quantified by RT-qPCR of patients without reflux (NR) and with reflux (R), as well as by their ages. * *p* < 0.05, ** *p* < 0.005.

Gene expression of fibulin 5 (FBLN-5) was significantly higher in R patients (NR = 50.949 ± 20.892, R = 100.240 ± 34.316, ** *p* < 0.005, Figure 12). Taking into account the age factor, a significant increase was observed in R < 50 patients (NR < 50 = 35.708 ± 19.596, NR > 50 = 66.191 ± 3.203, R < 50 = 108.948 ± 34.212, R > 50 = 89.789 ± 35.079, * *p* < 0.05, Figure 12).

**Figure 13:**
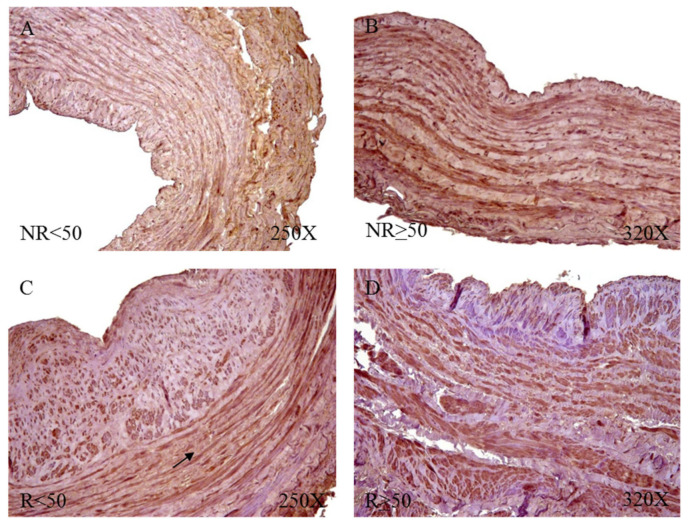
Protein expression of fibulin 5 in NR < 50 (**A**), NR ≥ 50 (**B**), R < 50 (**C**) and R ≥ 50 (**D**).

Protein expression showed how R patients had a higher percentage of reactivity (NR = 65.52%, R = 82.72%). We observed how patients R < 50 had the highest percentage of positive expression for FBLN-5 (NR < 50 = 38.46%, NR > 50 = 87.50%, R < 50 = 96.88%, R > 50 = 73.47%). Microscopic observation showed how FBLN-5 was present in the three tunics of the vein wall, with a similar intensity of expression in all groups. It was possible to point out how FBLN-5 was especially intense in smooth muscle bundles. This pattern was maintained in the patients with positive reactivity in the four study groups (Figure 13).

### 3.4. Young Patients with Venous Reflux Show Increased Levels of Fibrillin 1 Expression

**Figure 14:**
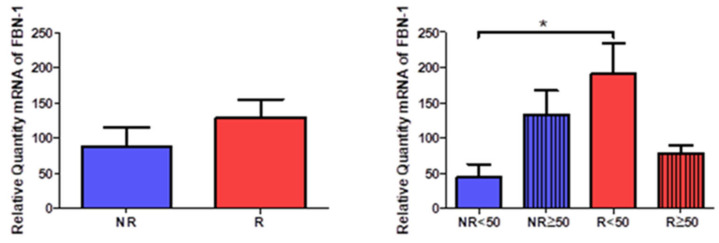
Expression levels of the gene for fibrillin 1 quantified by RT-qPCR of patients without reflux (NR) and with reflux (R), as well as by their ages. * *p* < 0.05.

The study of the genetic expression of fibril 1 (FBN-1) showed a tendency to increase in patients with venous reflux (NR = 87.971 ± 65.168, R = 128.337 ± 88.774, Figure 14). When observing their distribution according to the age of the patients, there was a statistically significant increase in R < 50 patients (NR < 50 = 43.365 ± 33.413, NR > 50 = 132.578 ± 59.427, R < 50 = 190.247 ± 98.191, R >50 = 76.745 ± 31.961, Figure 9).

When studying the protein expression of FBN-1, it was possible to observe how the R patients present a higher percentage of expression (NR = 55.17%, R = 85.19%), seeing how R < 50 have the highest percentage (NR < 50 = 30.77%, NR > 50 = 75.00%, R < 50 = 96.88%, R > 50 = 77.55%).

**Figure 15:**
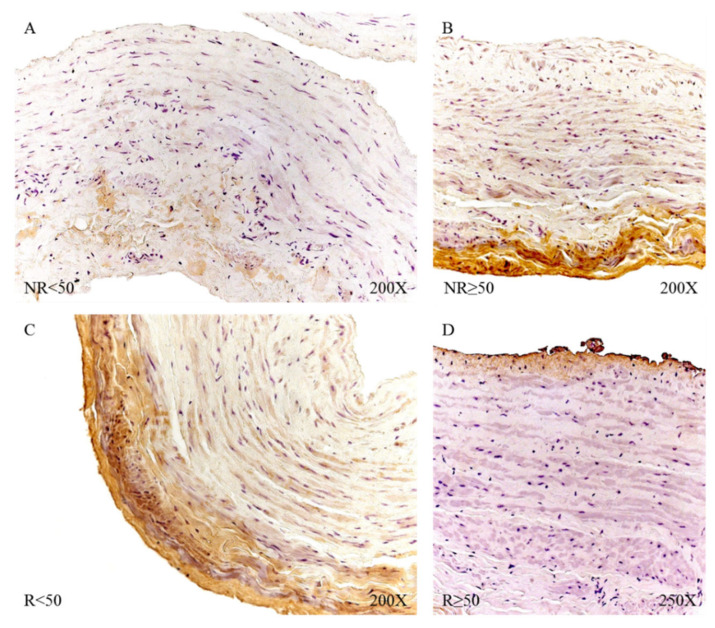
Fibrillin 1 protein expression images in patients NR < 50 (**A**), NR > 50 (**B**), R < 50 (**C**) and R > 50 (**D**).

Microscopic visualization of samples with positive immunoreactivity showed how FBN-1 in patients with NR < 50 was deposited in small accumulations in the transit zones between the tunica media and adventitial (Figure 15A). In the case of patients with NR > 50, said deposit was present throughout the adventitial layer, with an intensity of medium expression (Figure 15B). The R < 50 patients had great expression in the adventitial tunica that extended towards the median tunica of the vein wall (Figure 15C); in contrast, the R > 50 patients presented an expression in the venous endothelium with small deposits in the adventitial tunic (Figure 15D).

### 3.5. Young Patients with Venous Reflux Show Increased Expression of LOX and LOXL-1

**Figure 16:**
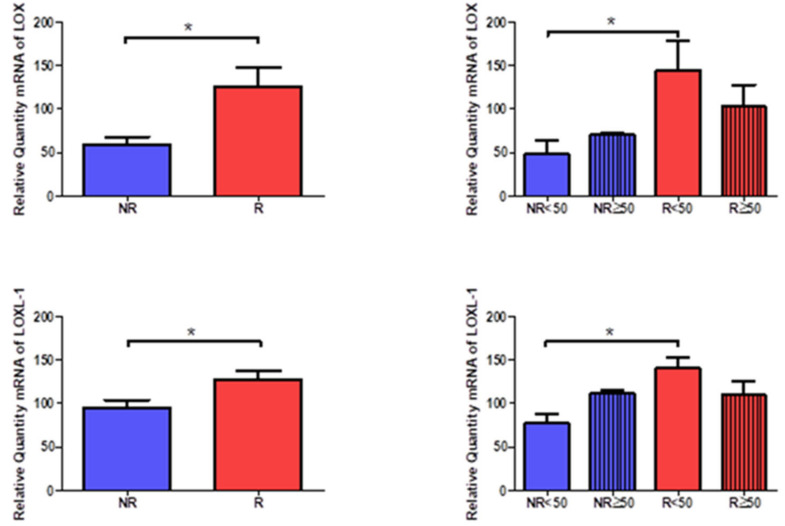
Expression levels of the gene for LOX and LOXL-1 quantified by RT-qPCR of patients without reflux (NR) and with reflux (R), as well as by their ages. * *p* < 0.05.

The gene expression of LOX and LOXL-1 showed statistically significant differences in relation to the presence of venous reflux, which were higher in venous reflux (LOX = NR = 59.612 ± 19.997, R = 125.459 ± 73.142, * *p* < 0.05, LOXL-1= NR = 94.593 ± 22.303, R = 126.588 ± 35.777, * *p* < 0.05, Figure 16). Taking into account the age factor, it was possible to describe how the levels of LOX and LOXL-1 were significantly higher in R < 50 patients compared to NR < 50. (LOX = NR < 50 = 48.672 ± 25.203, NR > 50 = 70.386 ± 2.360, R < 50 = 144.409 ± 84.643, R > 50 = 102.720 ± 56.867, LOXL-1= NR < 50 = 77.534 ± 18.338, NR > 50 =111.653 ± 5.864, R < 50 = 140.529 ± 31.767, R > 50 = 109.860 ± 36.016, * *p* < 0.05, Figure 16).

The protein study showed that the highest percentage of positive expression was detected in the groups with venous reflux for LOX and LOXL-1 (LOX = NR = 51.72%, R = 88.89%, LOXL-1 = NR = 62.07%, R = 86.42%). Taking age into account, the percentages for LOX and LOXL-1 were similar, even equal in the case of R < 50 patients (LOX = NR < 50 = 46.15%, NR > 50 = 56.25%, R < 50 = 96.88%, R > 50 = 83.67%, LOXL-1= NR < 50 = 61.54%, NR > 50 = 62.50%, R < 50 = 96.88%, R > 50 = 81.63%).

**Figure 17:**
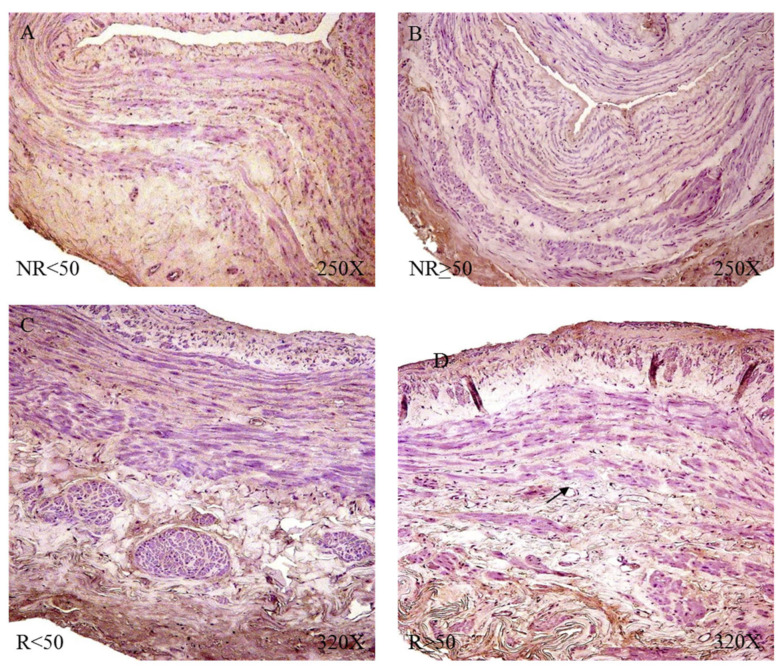
Protein expression of LOX in patients NR < 50 (**A**), NR ≥ 50 (**B**), R < 50 (**C**) and R ≥ 50 (**D**).

The microscopic study revealed that LOX was located in the intima and adventitial tunics of the vein wall, with the majority of its expression being in the myointimal part near the endothelium of the vein in the cases in which it was present and in the entire vein. Tunica adventitial was apparent in all patients. In patients R < 50, a slightly more intense expression was visualized in the medial and adventitial tunica between the muscle bundles (Figure 17).

**Figure 18:**
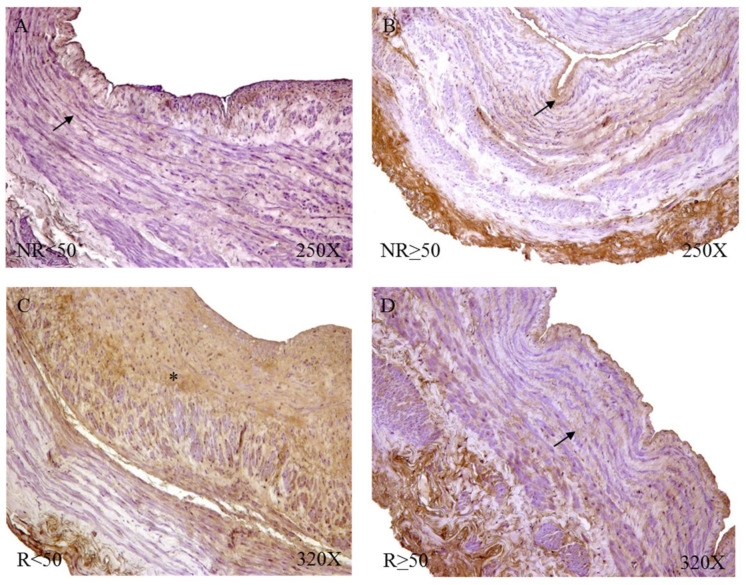
Protein expression of LOXL1 in patients NR < 50 (**A**), NR ≥ 50 (**B**), R < 50 (**C**) and R ≥ 50 (**D**).

LOXL-1 showed a different expression pattern than that presented by LOX. In the case of NR < 50 patients, LOXL-1 was located in the muscle bundles close to the tunica intima, as a slight protein expression in terms of its intensity (Figure 18A). Patients with NR > 50 had a presence of LOXL-1 in the tunica intima with a very marked endothelium as well as in the tunica adventitial. In this group of patients, it was possible to observe how LOXL-1 was visualized in the areas close to where the valve insertion occurs (Figure 18B). The R < 50 patients had a high expression in terms of intensity throughout the entire length of the vein wall, and a large deposit of said protein can be observed in the tunica intima in tune with the smooth muscle fibers (Figure 18C). In the case of patients R > 50, the expression was lower in terms of expression in the tunica media (Figure 18D).

## 4. Discussion

Previous studies described a restructuration of the elastic component in the venous wall of patients with VVs in comparison to healthy subjects [20]. Further, our results suggest that the elastic component and its main precursors are also different in VVs of patients with pathological venous reflux in comparison to those without reflux. Besides, there are some differences in younger patients (<50 years old) with venous reflux in comparison to those without venous reflux and elder patients with and without venous reflux. Our results are in agreement with previous studies in which young patients with venous reflux exhibited different molecular and tissue markers in comparison to older patients and those without venous reflux [13,15,21,22,23].

Venous reflux is relatively common in patients with CVeD [24]. As mentioned above, an advanced age is directly correlated with a greater risk to suffer from venous reflux [9]. Young patients with VVs seem to present an enhanced genetic susceptibility to suffer from CVeD, triggering the onset and development of venous reflux [25]. Our study suggests that neither the total number of elastic fibers, nor the diameter in the tunica intima and adventitia, vary between VVs of patients with reflux when comparing with those without reflux, although the total number of elastic fibers is significantly reduced in the media layer of patients with venous reflux. This fact may be indicator of an abnormal behavior of smooth muscle cells, probably with a boosted proteolytic activity [26], thereby reflecting the increased damage and alterations that patients with venous reflux are suffering. More interestingly, when we compare the total number of fibers in the venous wall according to the age, we observe that young patients with reflux show a significant increase in the elastic fibers and its diameter. These differences are in agreement to previous results observing that young patients with venous reflux exhibit an enhanced tissue remodeling, with a distinct pattern of matrix metalloproteases (MMP) activity and their endogenous tissue inhibitors of MMP (TIMPs) [13]. The enhanced elastic content in the vascular wall of young patients with reflux could indicate the need for greater dynamism in the environment of these areas of the venous wall. Interestingly, these changes are also observed in patients aged ≥ 50 without reflux, which may support the hypothesis that venous reflux may result in an accelerated aging of the vascular wall. Besides, some studies have described that the thickening of the intima layer in VVs is directly correlated to increased quantities of elastic material [27]. Our results may support this statement, as previous works have also observed an augmented thickening of this layer in patients with great saphenous vein reflux [28]. Additionally, Sansilvestri-Morel et al. reported that the changes of elastic components in the veins were correlated with those occurring in the skin [29]. Therefore, it is possible that the changes in the elastic components observed in young patients with venous reflux may accelerate the progression from of CVeD to CVI.

Conversely, elder patients with venous reflux show significant differences in the elastic content in comparison to elder subjects without reflux. As previous research has evidenced a direct correlation between elastin fibers in the adventitia with vein diameter at rest [30], it is logical to think that patients with venous reflux exhibit increased damage in the venous wall, with a reduced functional media layer probably related to the hypoxia associated to this condition [31]. Despite young patients without reflux display significant differences in the intima and adventitia layer of elastic fibers but not in the tunica media, it could mean that the exposure of this group to some pathological mechanisms is limited in comparison to young patients with reflux. However, over the years, the sustained pathological environment of CVeD may lead to important changes in the venous wall of these patients, and they may be at higher risk to suffer from venous reflux.

Furthermore, we detected significant differences in the expression of several precursors of elastic fibers. Tropoelastin (TE) is significantly upregulated in patients with venous reflux in comparison to patients without reflux, independently from the variable age. TE is the major structural unit of elastic fibers [32]. TE is produced within the cell, especially smooth muscle cells, and secreted to the ECM. There, it is assembled with FBLN4 and FBLN5, then fibrillin 1 and 2 stabilize microfibrils of TE and, eventually, LOX and LOXL1 drive to the cross-linking of TE and formation of mature elastic fibers [33]. The increased levels of TE as well as other components previously described in patients with venous reflux may indicate that an enhanced synthesis of elastic fibers may be a critical homeostatic response in venous reflux. In this sense, Pascual et al. [34] reported that TE was upregulated in young patients with VVs, but the levels of this component tend to diminish with aging. The presence of venous reflux seems to stimulate the synthesis of TE, independently from the age of the patient. It is of note that the enhanced expression of TE is mostly found in the intima and media layer, specially comparing young patients with and without reflux. These may be the consequence of the further dynamism in the venous wall described in patients with reflux.

FBLN5 but not FBLN4 is significantly upregulated in patients with venous reflux. In more detail, there are significative differences between young patients with and without reflux. Previous studies have argued that FBLN5 plays a pivotal role to maintain vessel integrity during various pathological insults [35]. Besides, it has been proposed as an endogenous inhibitor of the angiogenesis process [36]. It is possible that the expression of FBLN5 may result in some disparities in the local angiogenesis between both groups, although further studies are required in this sense. Moreover, fibrillin 1 is also differentially expressed in young patients with reflux in comparison to those without reflux.

Fibrillin-1 is mainly produced by smooth muscle cells and previous studies denoted an increase of fibrillin 1 in the veins and skin of patients with CVeD [29]. Furthermore, Bastos et al. [37] reported that fibrillin-1 showed a distinct expression in VVs in women aged over and under 50. This could be related to the fact that fibrillin 1 is an important modulator of transforming growth factor-β (TGF-β) [38], and previous studies have demonstrated an important dysregulation of this component related to a pathological aging process of the VV [39]. In this sense, young patients affected by venous reflux appear to display a different matrix remodeling than those without reflux. Finally, LOX and LOXL1 are two molecules with greater expression in patients with venous reflux. Our results suggest that LOX expression is significantly higher in aged patients with venous reflux than in those without reflux, whereas LOXL-1 presents higher expression in young patients with reflux in comparison to young patients without reflux. LOX is an important marker of vascular integrity, and its expression levels seems to be directly correlated with different tissue markers like α-smooth muscle cell actin and the proper TGF-β [40]. LOXL-1 also shows a differential expression pattern related to the aging process [34]. Hence, it seems that both LOX and LOXL-1 may be critical for supporting the vascular remodeling and dynamism necessary in patients with pathological reflux.

## 5. Conclusions

Young patients with venous reflux report notable differences with young patients without reflux and elder subjects with vein reflux, suggesting a greater dynamism and arterialization process of the venous wall in this group of patients.

## Figures and Tables

**Table 1:** Patients according to CEAP classification.

NR	C1	34.48 (10)	NR < 50	C1	30.76 (4)
				C2	69.24 (9)
	C2	62.07 (18)		C3	0.00 (0)
				C4	0.00 (0)
	C3	3.45 (1)	NR ≥ 50	C1	37.50 (6)
				C2	56.25 (9)
	C4	0.00 (0)		C3	6.25 (1)
				C4	0.00 (0)
R	C1	0.00 (0)	R < 50	C1	0.00 (0)
				C2	34.37 (11)
	C2	44.44 (36)		C3	53.13 (17)
				C4	12.50 (4)
	C3	48.15 (39)	R ≥ 50	C1	0.00 (0)
				C2	51.02 (25)
	C4	7.41 (6)		C3	44.89 (22)
				C4	4.09 (2)

Abbreviations: NR = absence of pathologic reflux; R = presence of pathologic reflux.

**Table 2:** Primary and secondary antibodies with dilution factors and protocol specifications in the immunohistological studies.

Antigen	Species	Dilution	Provider	Protocol Specifications
TE (Tropoelastin)	Rabbit (Polyclonal)	1:750	Dr. Mecham Washington University	--------------------
FBLN-4 (Fibulin 4)	Rabbit (Monoclonal)	1:250	Abcam (ab125073)	Citrate tampon in heat (pH = 6.0)
FBLN-5 (Fibulin 5)	Rabbit (Polyclonal)	1:1000	Abcam (ab202977)	--------------------
FBN-1 (Fibrillin 1)	Rabbit (Polyclonal)	1:100	Abcam (ab53076)	Triton 100 × 0.1% in PBS, 10 min
LOX (Lysyl oxidase)	Rabbit (Polyclonal)	1:500	Dr. Sommer CNRS-UMR	Glycine HCl, 30 min RT. 0.2% Hialuronidase, 30 min 42 °C
LOXL-1 (Lysyl oxidase-like 1)	Rabbit (Polyclonal)	1:250	Dr. Sommer CNRS-UMR	Glycine HCl, 30 min RT. 0.2% Hialuronidase, 30 min 42 °C
IgG (Rabbit)	Mouse polyclonal	1:1000	Sigma-Aldrich (RG-96/B5283)	--------------------

Abbreviations: PBS = phosphate buffered saline; RT = room temperature.

**Table 3:** Sequences of the primers and their binding temperatures in the RT-qPCR studies.

Gene	Sequence Fwd (5′→3′)	Sequence Rev (5′→3′)	Temp
*GADPH*	GGA AGG TGA AGG TCG GAG TCA	GTC ATT GAT GGC AAC AAT ATC CAC T	60 °C
TE (Tropoelastin)	GTG TAT ACC CAG GTG GCG TG	CGA ACT TTG CTG CTG CTT TAG	64 °C
FBLN-4 (Fibulin 4)	GTC TTG GAC ATG CCA GGA TTA	TGG AGA TCG TGG GAT AGT TTG	60 °C
FBLN-5 (Fibulin 5)	GTC TTG GAC ATG CCA GGA ATA	TGG AGA TCG TGG GAT AGT TTG	58 °C
FBN-1 (Fibrillin 1)	GGT GAA TGT ACA AAC ACA GTC AGC A	ATA GGA ACA GAG CAC AGC TTG TTG A	60 °C
LOX (Lysyl oxidase)	GCA GAT GTC AGA GAT TAT GAT CA	ATC GCC TGT GGT AGC CAT AGT	60 °C
LOXL-1 (Lysyl oxidase-like 1)	GCA CCT CTC ATA CCC AGG GC	TGG CAG TCG ATG TCC GCA T	60 °C

**Table 4:** Number of elastic fibers determined by orcein. Mean ± SD.

Groups	Total	Intima	Media	Adventitia
NR	58.764 ± 12.663	26.127 ± 14.384	26.833 ± 1.941	30.714 ± 17.783
R	53.000 ± 39.711	15.636 ± 15.609	20.000 ± 5.633	27.250 ± 20.908
NR < 50	46.800 ± 2.961	7133 ± 0.777	27.667 ± 2.517	12.000 ± 1.000
NR ≥ 50	63.250 ± 11.925	33.250 ± 9.099	26.000 ± 1.000	44.750 ± 4.349
R < 50	114.667 ± 5.508	39.667 ± 4.726	24.750 ± 3.615	46.500 ± 9.411
R ≥ 50	29.875 ± 1.808	6.625 ± 1.188	15.250 ± 1.832	8000 ± 1.069

Abbreviations: NR = absence of pathologic reflux; R = presence of pathologic reflux.

## Data Availability

The data used to support the findings of the present study are available from the corresponding author upon request.
